# Number of Matriculates in the Atlanta Medical College

**Published:** 1856-09

**Authors:** 


☞ The number of Matriculates in the Atlanta Medical College, for 1856, is 105.

